# Unusual Findings With Imaging-Guided Fine Needle Aspiration

**DOI:** 10.1155/crip/7005824

**Published:** 2025-04-24

**Authors:** Robert Pei, Shane M. Woods, Brant G. Wang

**Affiliations:** ^1^University of Virginia School of Medicine Inova Campus, Falls Church, Virginia, USA; ^2^Department of Pathology, Inova Fairfax Hospital, Falls Church, Virginia, USA; ^3^Department of Pathology, Georgetown University Medical Center, Washington, DC, USA; ^4^Department of Pathology and Immunology, Baylor College of Medicine, Houston, Texas, USA

**Keywords:** barium crystals, fine needle aspiration, polarized microscopy, starch

## Abstract

For deep-seated lesions, fine needle aspiration (FNA) under imaging guidance may be crucial to secure material for definitive diagnosis and further management. Rarely, components other than cells and tissue fragments may be visualized upon microscopic scrutiny following biopsy. These findings may lead to confusion in diagnosis. We describe two cases in which refractile foreign materials caused diagnostic challenges. The material in the first case turned out to be barium crystals left at a prior procedure for imagining study. The material in the second case was most likely starch-based material the patient aspirated or inhaled. These two cases highlight the importance of attention to details and judicious use of polarized microscopy.

## 1. Introduction

For deep-seated lesions, fine needle aspiration (FNA) under imaging guidance may be crucial to secure material for definitive diagnosis and further management. Rarely, components other than cells and tissue fragments may be visualized upon microscopic scrutiny following biopsy. These findings may lead to confusion in diagnosis.

Polarized microscopy has been used to identify materials on FNA samples, including foreign body materials (retained surgical textile, cosmetic dermal fillers, talc, silica, and chromium), parasite, calcium oxalate, amyloid, monosodium urate, calcium pyrophosphate dihydrate, galactocele protein, and tyrosine [1–18]. It can also be used to identify chondroid tissue in pleomorphic adenoma [19]. Crystals and crystalloids in cytological material were described in an elegant review paper [20].

We describe two cases in which refractile foreign materials caused diagnostic challenges.

## 2. Case Presentation #1

**Figure 1:**
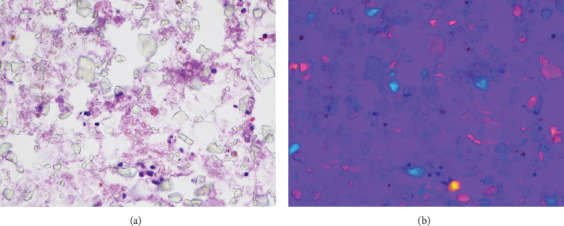
(a) Endoscopic ultrasound–guided FNA of thickened gastric wall (Diff-Quik x200). Finely granular crystals of varied sizes and shapes mimic calcification. (b) Endoscopic ultrasound–guided FNA of thickened gastric wall. Under polarized microscope, the crystals are refractile parallel and perpendicular to the polarizer, yellow and blue, respectively (Diff-Quik x200).

A female in her early 50s underwent emergent laparoscopic closure of a gastric perforation at an outside hospital. Seven weeks later, she presented at the emergency department for worsening nausea and vomiting. Computed tomography (CT) findings were suspicious for gastric outlet obstruction. Endoscopy revealed gastric outlet obstruction, gastritis, irregular mucosa in both the antrum and body, and findings highly suspicious for linitis plastica. Two endoscopic studies were limited due to persistent gastric contents in the stomach. Biopsies were taken but were not diagnostic. She was transferred to our institution for further management of gastric outlet obstruction and suspected gastric malignancy. Endoscopic biopsy was unsuccessful due to obscuring white material. Endoscopic ultrasound–guided FNA (EUS-FNA) of the gastric wall was then attempted. Diff-Quik-stained direct smear showed light yellow translucent crystals resembling calcification throughout (Figure 1). Under polarized microscope, the crystals were refractile both parallel and perpendicular to the analyzer (Figure 1). The FNA revealed only barium crystals with no diagnostic material for malignancy. A mucosal biopsy 3 days later showed rare, atypical cells followed by another biopsy showing benign findings. The subsequent biopsy and peritoneal washing a week later confirmed the diagnosis of diffuse-type gastric adenocarcinoma with peritoneal involvement. The patient expired 15 months after the diagnosis of malignancy and chemotherapy.

## 3. Case Presentation #2

**Figure 2:**
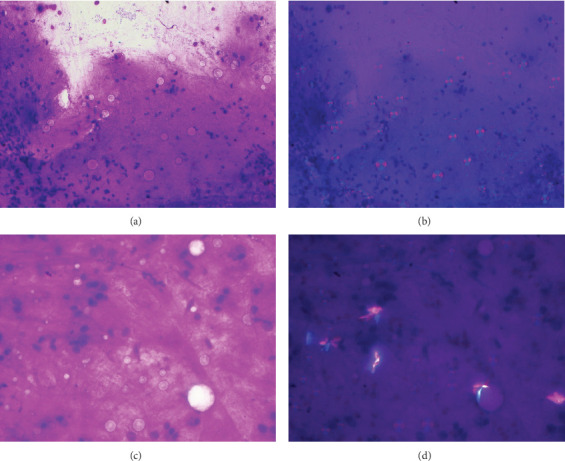
(a) Navigational FNA of a lung nodule (Diff-Quik x200). Microspheres mimic *Blastomyces* fungi. (b) Under polarized microscope, the microspheres are refractile at a 45° angle to the polarizer. Maltese cross present. Diff-Quik stained, original x200. (c) Microspheres with some of which are degraded. Diff-Quik stained, original x400. (d) Degraded microspheres under polarized microscope. Diff-Quik stained, original x400.

A male in his mid-60s with history of end-stage renal disease on hemodialysis, hypertension, and Type II diabetes mellitus underwent workup for a kidney transplant. An incidental 1.3-cm ground-glass nodule of the left lower lobe was identified on CT imaging. Electromagnetic navigation bronchoscopy–guided FNA of this nodule showed microspheres of up to 30 *μ*m in diameter, reminiscent of compact discs, donuts, or broad-based buds seen in *Blastomyces dermatitidis* infection; however, under polarized microscope, the microspheres were refractile at a 45^o^ angle to the polarizer (Figure 2). Transbronchial biopsy of the lung nodule showed minute fragments of tissue suspicious for low-grade B-cell lymphoma or mucosa-associated lymphoid tissue (MALT) lymphoma. The microspheres seen on FNA were not identified on the limited cell block material. The patient was scheduled for a 6-month follow-up but was lost to follow-up.

## 4. Discussion

Identifying the materials in these two cases was a challenge. In the first case, the barium crystals left at previous procedures were thought to be calcification of various causes, such as dystrophic, gastric mucosal calcification associated with hypercalcemia, stasis to flow, and tumor calcification [21]. However, with polarized microscopy, it became apparent that these polarizable crystals were barium particles instead of calcifications, as the aforementioned calcifications do not polarize [22–24]. This conclusion was corroborated by the gastroenterologist who performed the FNA procedure upon further discussion. Note that no attempt was made to confirm the diagnosis by energy dispersive x-ray spectroscopy [22, 24]. The patient's gastric outlet obstruction contributed to the delay of barium clearing. Immunostains of the FNA sample to exclude malignancy were not attempted due to lack of cells on direct smear or cell block.

In the second case, the refractile microspheres confounded cursory identification. Under nonpolarized light, these microspheres were originally thought to be broad-based buds seen in *Blastomyces dermatitidis* infection (Figure 2a). However, there was no background necrosis or granulomatous inflammation typical of such findings. Furthermore, the noted resemblance to compact disc or donut was unusual for yeast. With polarized microscopy, the birefringent nature of these microspheres became apparent. The spherical shape with maltese cross, noted color, and size most closely matched with starch [25–28]. On further inquiry, it was noted that the patient was a construction worker on disability. These microspheres were most likely starch-based foreign materials such as starch microspheres in medications, since the patient had been on multiple medications, or he might simply had aspiration of starch food into his left lower lobe. Since he was a construction worker, inhalation of starch-containing construction material could not be excluded. These microspheres were not pulmonary surfactant stored in lamellar bodies of Alveolar Type II cells since they were too large for surfactant [29]. The spherical shape was not a match for other materials detected under polarized microscope, such as talc, silica, chromium, calcium oxalate, amyloid, monosodium urate, calcium pyrophosphate dihydrate, and tyrosine. Contamination during sample processing was ruled out, as some microspheres showed degradation and were only visible within the FNA material, not outside the direct smear (Figure 2c,d). This degradation phenomenon was similar to that described in another case [30]. The microspheres were less likely products of a neoplastic process. Immunostains attempted on transbronchial biopsy material of the lung nodule raised the suspicion of a low-grade B-cell lymphoma. Although, theoretically, Russell bodies can be present as microspheres, there is no literature reporting Russell bodies as birefringent refractile microspheres.

These two cases highlight the importance of attention to details and judicious use of polarized microscopy. Besides the more common findings of calcium oxalate, amyloid, monosodium urate, and calcium pyrophosphate dihydrate, one needs to consider foreign body materials in the right clinical settings.

## Data Availability

The data that support the findings of this study are available on request from the corresponding author. The data are not publicly available due to privacy or ethical restrictions.
